# Effects of experimental cervical spinal cord injury on peripheral adaptive immunity

**DOI:** 10.1371/journal.pone.0241285

**Published:** 2020-10-30

**Authors:** Antigona Ulndreaj, Apostolia Tzekou, Ahad M. Siddiqui, Michael G. Fehlings

**Affiliations:** 1 Division of Genetics & Development, Krembil Research Institute, University Health Network, Toronto, Ontario, Canada; 2 Institute of Medical Science, University of Toronto, Toronto, Ontario, Canada; 3 Department of Surgery, University of Toronto, Ontario, Canada; 4 University of Toronto Spine Program, University of Toronto, Ontario, Canada; Imperial College London, UNITED KINGDOM

## Abstract

Adaptive immunity is critical for controlling infections, which are a leading cause of morbidity and mortality in patients with spinal cord injury (SCI). In rats and mice, compromised peripheral adaptive immune responses, as shown by splenic atrophy and lowered frequencies of peripheral lymphocytes, were shown to result from high-level thoracic SCI. However, whether cervical SCI, which is the most common level of SCI in humans, impairs adaptive immunity remains largely unknown. In the present study, we induced cervical SCI in rats at the C7/T1 level by clip compression and looked at changes in peripheral adaptive immunity at 2-, 10- and 20-weeks post-injury. Specifically, we quantified changes in the frequencies of T- and B- lymphocytes in the blood and the mandibular and deep cervical lymph nodes, which drain the cervical spinal cord. We also assessed changes in serum IgG and IgM immunoglobulin levels, as well as spleen size. We found a significant decline in circulating T- and B- cell frequencies at 10 weeks post-SCI, which returned to normal at 20 weeks after injury. We found no effect of cervical SCI on T- and B- cell frequencies in the draining lymph nodes. Moreover, cervical SCI had no effect on net spleen size, although injured rats had a higher spleen/body weight ratio than sham controls at all time points of the study. Lastly, IgG and IgM immunoglobulin declined at 2 weeks, followed by a significant increase in IgM levels at 10 weeks of injury. These data indicate that cervical SCI causes a significant imbalance in circulating lymphocytes and immunoglobulin levels at 2 and 10 weeks. As we discuss in this article, these findings are largely in line with clinical observations, and we anticipate that this study will fuel more research on the effect of adaptive immunity on SCI recovery.

## Introduction

Cervical spinal cord injury (SCI) is the most frequent type of traumatic SCI seen in the clinic [1]. This level of injury typically causes more severe deficits and is associated with higher healthcare costs than lower level injuries [2]. The immune system is a critical component of the pathophysiology of SCI [3], and treatments that modulate the immune response hold great clinical promise [4]. In fact, the only currently approved treatment for SCI, methylprednisolone sodium succinate (MPSS), is a glucocorticoid with widespread immunosuppressive activity. However, MPSS increases the susceptibility to infections and its neuroprotective effects for patients with SCI are generally small [5]. Thus, immunomodulatory therapies with a better benefit-to-risk profile are needed, rendering further research into the immune response of SCI pathophysiology necessary.

Some of the biggest challenges in the quest for better treatments for SCI are the dual nature of the immune responses elicited in the course of the disease [6], coupled with the effect of the SCI anatomical level on the immune response [7–10]. Specifically, on the one hand, excessive innate and adaptive immune responses attack the neural tissue, thereby propagating the damage caused originally by the injury [3]. Whereas, on the other hand, suppressed peripheral (herein referred to as the response outside the spinal cord) adaptive immune responses against common pathogens render patients with SCI more susceptible to chronic infections. Infections are a major complication in SCI as they constitute the leading cause of mortality [11–13] and are associated with impaired neurological recovery post-injury [7, 11]. To counter this issue, vaccination has been suggested as a strategy to protect patients with SCI from infections [14]. However, as vaccination efficacy depends on a functional adaptive response, understanding the status of the adaptive immune response following SCI at various levels could expedite the design of effective vaccination strategies for patients.

We have previously shown that cervical SCI results in disturbed peripheral adaptive immune responses in the spleen of rats [15] and blood of humans [16]. Others have shown dysregulation of peripheral adaptive immunity in the spleen [10], blood [17] and lymph nodes (LNs) [9] in experimental and clinical thoracic SCIs. However, no study has investigated the effect of cervical SCI on adaptive immunity in major peripheral immune organs other than the spleen, and across various time points of the disease. This is a critical gap, given that the peripheral immune response affects the degree of inflammation within the spinal cord [18] and the response to microbial infections [7, 19], which collectively, deteriorate neurological recovery and quality of life in patients with SCI. To address this gap, we profiled changes in cells of adaptive immunity (T- and B- cells and CD4, CD8 T- cell subsets) located in the blood and LNs. We also quantified the level of serum IgG and IgM immunoglobulin and measured changes in spleen size. Consistent with our previous study in a C7/T1 rat SCI model [15], all metrics were quantified at three clinically-relevant time points of SCI –2, 10 and 20 weeks after injury–representing the subacute, early chronic and late chronic phases of injury, respectively.

In thoracic SCI, systemic adaptive immune responses are impaired in an injury-level dependent fashion, whereby higher level injuries cause significant immune suppression as indicated by a smaller spleen and reduced lymphocyte frequencies in the blood, spleen and LNs [7, 9, 10]. Hence, we hypothesized that injury at the cervical level would also suppress peripheral adaptive immunity, resulting in lowered T- and B- cell frequencies in blood and LNs, as well as reduced immunoglobulin levels and smaller spleen size. Our data revealed that immune compromise after cervical SCI is not absolute; rather, cervical SCI has dynamic effects on the status of the peripheral immune system depending on the tissue examined and on the time after injury. In particular, our data indicate that cervical SCI causes depression of adaptive immunity at 2 and 10 weeks after cervical SCI. However, these effects normalized by 20 weeks after cervical SCI.

## Materials and methods

### Surgical procedures study groups and tissue collection

A total of 111 adult, female Wistar rats (8 weeks old, 250–300 g, Charles River) were used for this study. All experiments involving animal use were prospectively approved by the University Health Network Animal Care Committee and experiments were carried out in strict accordance with approved protocols. All possible efforts were made to minimize animal suffering and the number of animals used in the study. Surgical procedures were performed using an operating microscope with rats anesthetized by 2% isoflurane delivered in a 1:1 mixture of O_2_/N_2_O. Sham animals underwent a C7-T1 laminectomy; muscles were sutured, and skin was closed with wound clips immediately, leaving the exposed spinal cord intact. The injured group received an injury for 60 seconds using a 35 g modified aneurysm clip, which was applied extradurally at the C7/T1 level, as previously established [20]. All animals were given a subcutaneous injection of 1 mL of buprenorphine (0.05 mg/kg) and 5 mL saline immediately, and subsequently twice a day for 3 days, after surgery.

After the injury or laminectomy alone, the animals were kept in the animal facility of the Krembil Research Institute where food and water were provided ab libitum. Manual bladder expression was performed three times a day. Animals that showed signs of infection were excluded from the study. When appropriate, naïve groups were introduced; these were age-matched rats that did not undergo any surgical procedure or standard postoperative care.

Tissues of interest were collected at 2, 10 and 20 weeks post injury (p.i.). Prior to euthanasia, animal body weight was recorded. Animals were deeply anaesthetized using isoflurane. Next, blood was collected by cardiac puncture followed by transcardial perfusion with phosphate buffered saline (PBS) alone, or PBS and 4% paraformaldehyde (PFA) in PBS, for the collection of fresh or fixed tissues, respectively. LNs were collected from animals perfused with PBS, whereas spleens were collected from PFA-perfused animals. Spleens were weighed to assess changes in spleen size for all study groups.

In ensuring minimal variability in our observations, animals in the sham and SCI groups of all time points were purchased in one batch and surgeries were performed consecutively by one experimenter within one week. The animals were randomly assigned to groups 2, 10 and 20 weeks and sacrificed at the respective time points. Animals of the same time point were sacrificed within two-three days, according to the time point of their survival based on their injury date. Since the 2-week time point for spleen weight assessment showed larger variability, we added 3 more animals in each group (sham and SCI) from surgeries that were performed by a second experimenter. All naïve animals were purchased in one batch, and they were sacrificed at respective time points based on their age, to match the age of SCI and sham animals at 2-, 10- and 20-weeks post operation, respectively. Naive animals were purchased at a later time point and their housing in the animal facility did not overlap with the SCI and sham rats, although they were placed in the same room as the rats in the SCI and sham group.

### Locating lymph nodes (LNs) that drain the spinal cord

The assessments in this section were conducted in rats without SCI. Briefly, while rats were under anesthesia by 2% isoflurane, muscle retraction and a two-level laminectomy at C7-T1 were performed. Then, using a 25G needle in a single pass, a small hole was made in the dura mater. To minimize cerebrospinal fluid (CSF) pressure due to the intrathecal (i.t) dye infusion, some CSF was allowed to leak (for approximately30 seconds) prior to dye administration. Next, a catheter (size 0.8Fr, Instech) was inserted through the hole supplying 30μl of india ink (VWR) in the subarachnoid space (SAS) over 2 minutes. Special care was taken to minimize back flow of the dye by further reducing its flow when necessary. However, during these manipulations some dye leaked into the surrounding muscles in most cases, which prompted us to introduce two control groups; a) animals that received a laminectomy where india ink was diffused in the surrounding muscle tissue alone, and b) animals that received a laminectomy but did not receive any dye. In both control groups the dura remained intact. Following the above procedure, muscles were sutured in layers, the skin was closed with wound clips and all animals were given a subcutaneous injection of 1 mL of buprenorphine (0.05 mg/kg) and 5 mL saline immediately, and subsequently every 12 hours, until sacrifice. At 4, 8 and 24 hours following dye infusion (*N* = 3 animals/ dye-administered group/time point, *N* = 1 for naïve group/time point), the animals were deeply anaesthetized by an overdose of isoflurane and perfused with ice cold PBS. Following dissection, draining LNs were identified based on positive staining by india ink, as observed grossly.

### Assessment of immune cell phenotypes in lymphoid organs and blood after cervical SCI

Blood and LNs were collected from rats with cervical SCI or sham injury at 2-, 10- and 20-weeks p.i and single cells were stained by fluorescent antibodies, as detailed below, to quantify % cell frequencies of T- lymphocytes and CD4^+^, CD8^+^ subpopulations, and of B- lymphocytes.

Blood was collected by cardiac puncture into an ethylenediaminetetraacetic acid (EDTA) coated tube (BD). Next, the samples were diluted 1:10 with red blood cell lysis buffer (0.1 mM EDTA, 10 mM KHCO_3_, 150 mM NH_4_Cl) for 5 min at room temperature (RT) and subsequently centrifuged at 2,000 rpm for 5 minutes. The remaining white blood cells were washed once in 2% fetal bovine serum (FBS) in PBS. The number of viable cells was quantified with Trypan Blue and 10^6^ viable cells were allocated per tube and incubated with the FcγR blocker (Innovex) for 20 minutes on ice. The samples were washed and subsequently stained for 30 minutes at 4°C with fixable viability dye (eFluor 780, Life Technologies, according to manufacturer’s instructions) followed by antibody staining. Fluorescent antibodies were grouped in two cocktails, as described below (all from BD Biosciences): Cocktail 1: CD45-FITC (leukocytes), CD3-PE (T- lymphocytes), CD4-PECy7 (T_helper_ cells), and CD8a-V450 (T_cytotoxic_ cells); or cocktail 2: CD45-FITC (leukocytes), CD45RA-V450 (B- lymphocytes). A LSR II flow cytometer was used for data acquisition, where at least 10,000 events of live cells were collected. Gating of positive populations was performed based on isotype-matched antibody controls (all from BD Biosciences). FlowJo V10 (Trestar) was used for data analysis and visualization.

LNs of interest were dissected out immediately after the animals were perfused with ice cold PBS and transferred to 2% FBS in PBS. Subsequently, the tissues were dissociated into single cells using a 70-μm nylon mesh cell strainer (Fisher). Next, red blood cells were lysed, and the remaining white blood cells were counted, fluorescently stained and analyzed, as described above.

### Quantification of the total level of serum IgG and IgM immunoglobulin

Blood was collected by cardiac puncture in deeply anaesthetized animals and allowed to clot for 30 minutes in RT. Samples were centrifuged for 10 minutes at 2,000 rpm and serum was collected and stored in -80°C until use for enzyme-linked immunosorbent assay (ELISA). To determine total levels of IgG and IgM antibodies, a sandwich ELISA was developed in house, as previously described (15). Briefly, ELISA plates were coated overnight at 4°C with 2 μg/ml of rabbit anti rat IgG- or goat anti rat IgM- capturing antibody (both from Abcam). Plates were then washed once with PBS and blocked with 4% goat serum in PBS for 2 hours at RT. Serum samples were diluted 1: 20,000 and 1:1,000,000 in blocking buffer for detection of IgM and IgG immunoglobulin, respectively, which then were incubated for 4 hours at RT. Polyclonal rat IgG (Sigma) and monoclonal rat IgM (Invitrogen) antibodies with known concentrations were used in serial dilutions to create appropriate standard curves that covered a detection range of 1 μg/ml—0.03 ng/ml. Duplicates of samples were in separate plates, whereas each plate had a duplicate standard curve. Following washes, horseradish peroxidase (HRP)-tagged anti-rat IgG or IgM antibody (both from Thermo Scientific) was incubated for 30 minutes at RT. Using Prism 6 (Graph Pad) software, a 4PL nonlinear curve fit was applied to the standard curve values, based on which, levels of IgG and IgM antibodies were determined in serum samples.

### Statistical analysis

SPSS (IBM) and Prism 6 (Graph Pad) software packages were used for statistical analyses. Data that met the criteria for parametric statistical analysis were subjected to parametric tests (Independent t-test for two groups, and analysis of variance [ANOVA] for more than two groups of samples with appropriate post hoc tests). Non-parametric statistical analyses (Mann-Whitney for two groups, and Kruskal Wallis test for more than two groups) were performed for non-parametric data. Serum immunoglobulin data were log10—transformed for variance stabilization prior to statistical analyses. All data are graphed as mean ± standard error of mean (SEM). The significance level of all analyses was set to *p* ≤ 0.05.

## Results

### Mandibular and deep cervical LNs drain the spinal cord at the C7/T1 level

**Fig 1:**
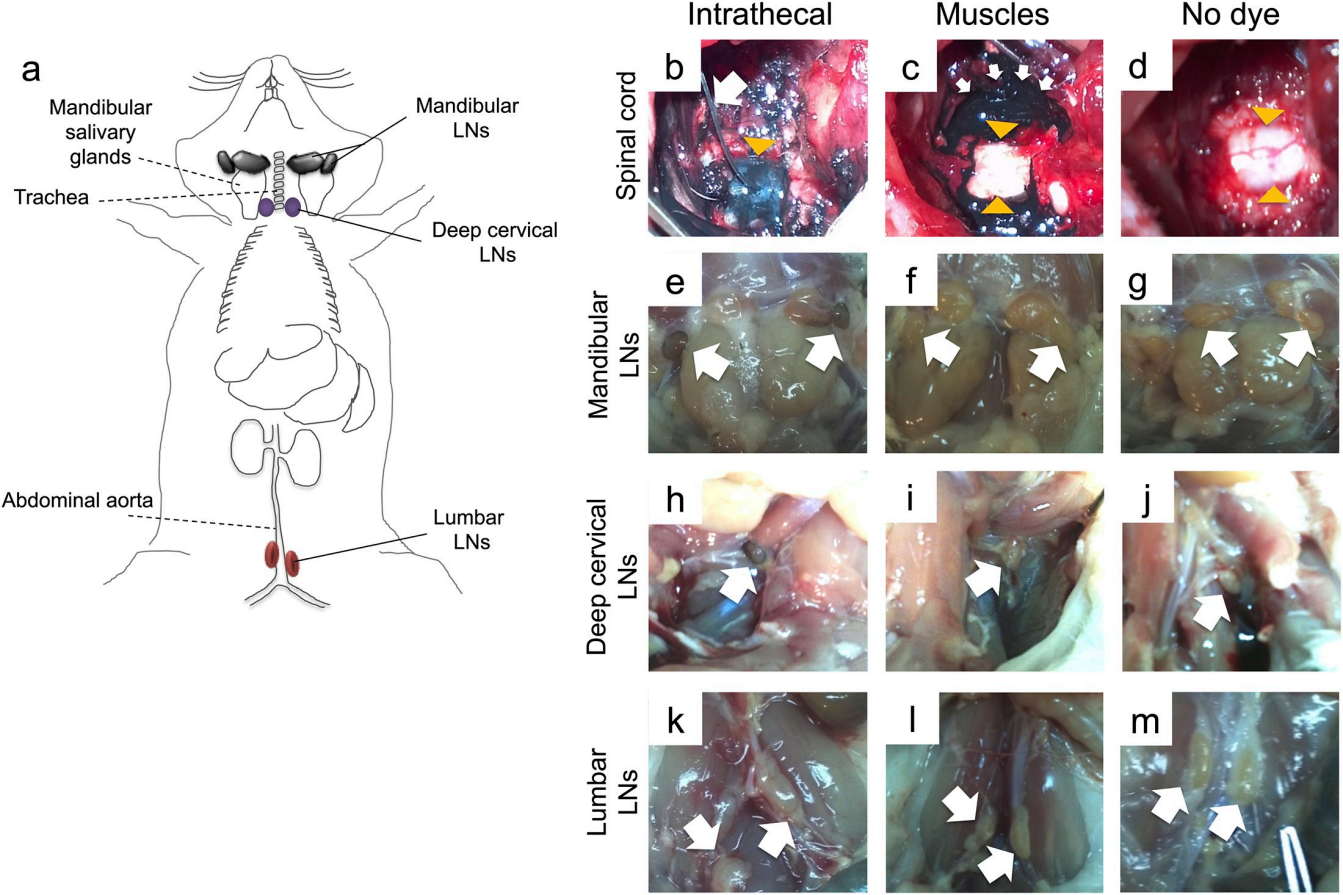
Locating LNs that drain the cervical spinal cord following intrathecal infusion of india ink. (a) Schematic drawing of rat LNs assessed macroscopically (solid lines) and respective anatomical landmarks (dotted lines). Mandibular LNs, herein referred to as mandibular and accessory mandibular LNs collectively [21], are located adjacent to and rostromedially from the sublingual and mandibular salivary glands. Cranial deep cervical LNs (referred to as deep cervical LNs) are dorsal to the trachea, and lumbar aortic LNs (here referred to as lumbar LNs) are adjacent to the abdominal aorta. (b) India ink was infused intrathecally by a catheter, which was inserted through a small hole in the dura at the C7/T1 level. The white arrow shows the catheter and the yellow arrowhead indicates the spinal cord, which is stained by the dye below the dura. (c) In control rats, the dura remained intact and the dye was allowed to diffuse in the surrounding muscles (white arrows), leaving the spinal cord unstained (yellow arrowheads). (d) In the third group, which received no dye, both the spinal cord (arrowheads) and muscles were unstained. (e-j) (e) Mandibular LNs and (h) deep cervical LNs of rats receiving intrathecal dye were consistently stained blue/black, but these LNs remained unstained in the (f, i) muscle control and the (g, j) no dye groups. (k-m) Lumbar LNs remained unstained in all groups.

Draining LNs were identified macroscopically based on positive staining by india ink, at 4, 8- or 24-hours following dye infusion. At all time points, mandibular and deep cervical LNs (Fig 1a) stained positive in animals receiving india ink into the SAS of the C7/T1 level (Fig 1b, 1e and 1h), but remained negative in the animals receiving india ink in the surrounding muscles (Fig 1c, 1f and 1i) and in the animals that received no dye (Fig 1d, 1g and 1j). Lumbar LNs remained unstained in all groups (Fig 1k, 1l and 1m) at all time points.

Taken together, these data indicate that mandibular and deep cervical LNs receive CSF drainage from the C7-level spinal cord. Lumbar LNs do not drain the C7-level.

### T- and B- cell frequencies in CSF draining LNs were unaltered following cervical SCI

**Fig 2:**
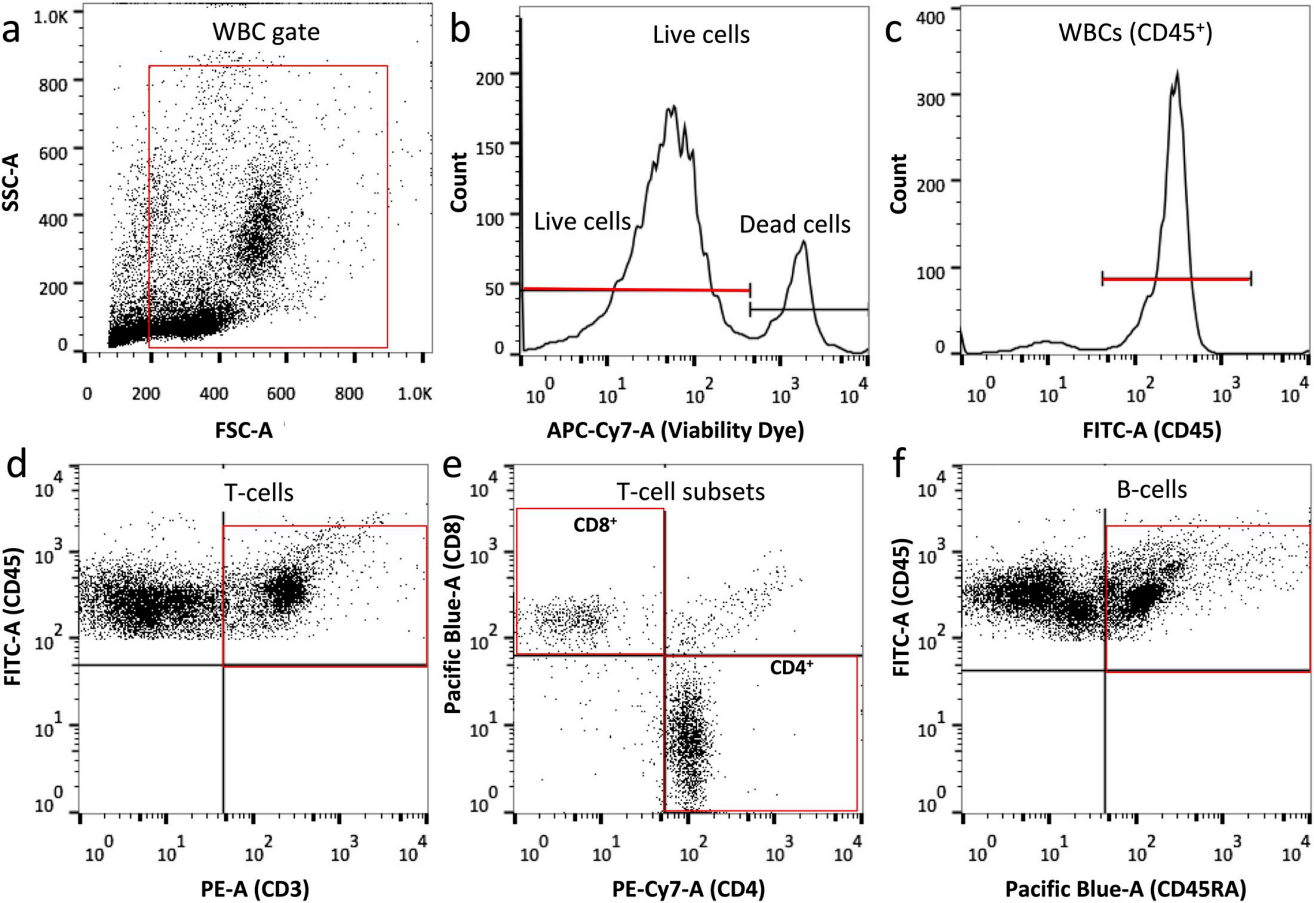
Gating strategy for T-, B- cells and CD4+, CD3+ T-cell subsets in blood and LNs. (a) Representative FSC/SSC dot plot diagram of blood cells. Red box indicates selection of white blood cells (WBCs) and exclusion of debris based on size and granularity. (b) Histogram indicating selection of live WBCs. (c) Histogram showing selection of WBCs out of live cells selected in (b), based on expression of CD45. (d) Selection of T-cells out of total CD45+ live cells, based on expression of CD3. (e) Selection of CD8+ and CD4+ T-cell subsets out of total T- cells as shown by red boxes. (f) Identification of B- cells out of total CD45+ live cells, based on expression of B- cell marker CD45RA. A similar gating approach was used for WBCs in LNs.

**Fig 3:**
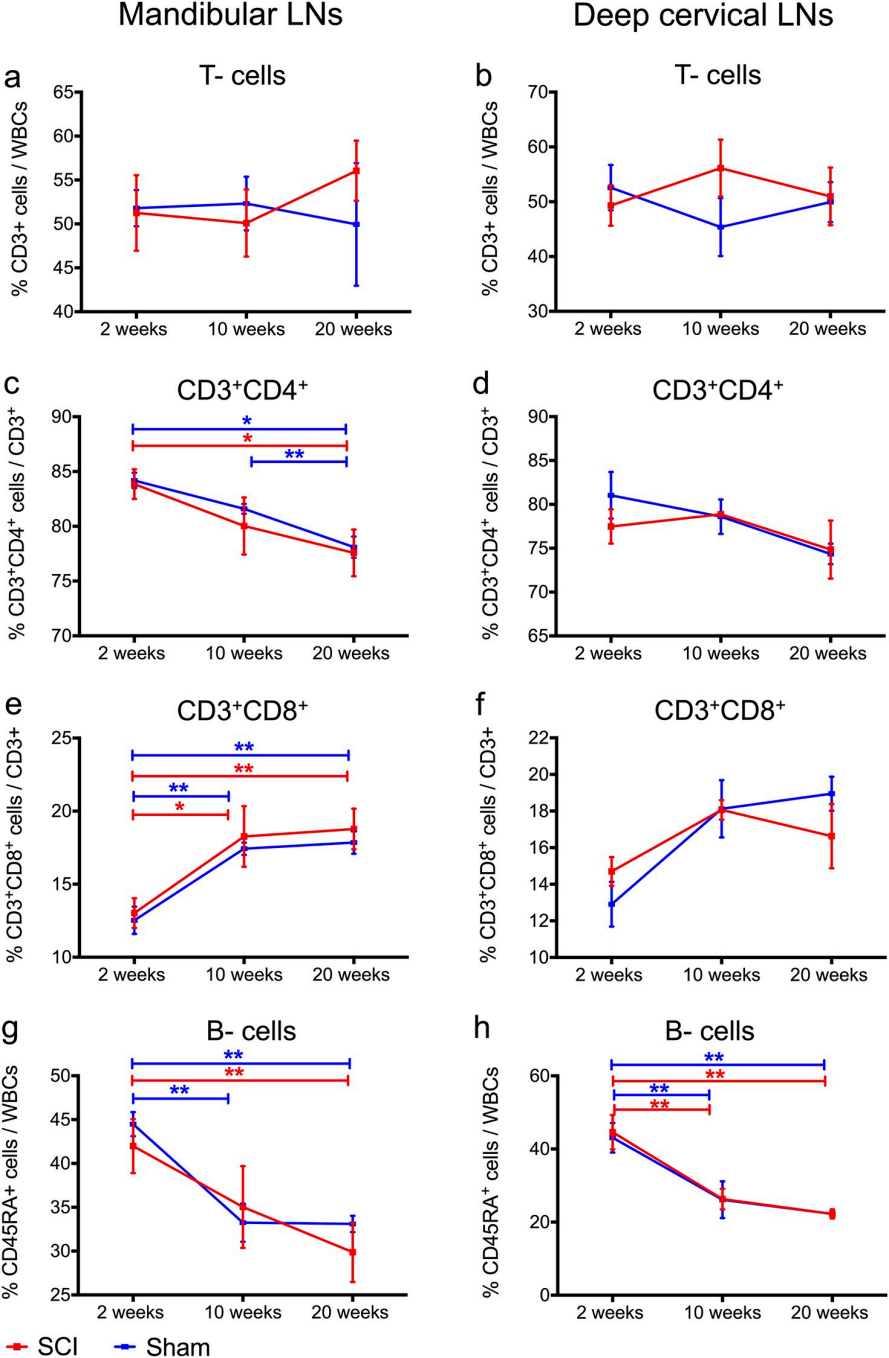
No significant changes in lymphocyte frequencies in the mandibular and deep cervical LNs following cervical SCI. (a-b) T-cell frequencies were similar between rats with cervical SCI and sham injury in both LN types, and these frequencies did not change with timing after injury. (c-d) CD4^+^ T-cell frequencies were similar between rats with cervical SCI and sham injury in both LN types. (c) In mandibular LNs, CD4^+^ T-cells decreased significantly with time post injury in both injury groups, and post-hoc tests revealed a significant difference from 2 to 20 weeks for the cervical SCI and sham group, and from 2 to 10 weeks for the sham group. (e-f) CD8^+^ T-cell frequencies were similar between cervical SCI and sham groups in both LN types. (e) In mandibular LNs CD8^+^ T- cells increased significantly with time post injury in rats with cervical SCI and sham injury, especially from 2 to 10 weeks and from 2 to 20 weeks post injury. (g-h) B- cell frequencies were similar between the cervical SCI and sham groups in both LN types. In addition, both groups showed time-dependent declining B- cell frequencies. (g) In mandibular LNs, B-cell frequencies dropped from 2 to 20 weeks for sham and cervical SCI rats, and from 2 to 10 weeks for shams. (h) In deep cervical LNs, B-cell frequencies declined from 2 to 10 weeks and from 2 to 20 weeks for both injury groups. Differences between the sham and cervical SCI group at each time point were assessed by Mann-Whitney tests, whereas time-dependent changes within each injury group were assessed by one-way ANOVA (sham) and Kruskal Wallis (cervical SCI) with Bonferroni or Mann-Whitney post-hoc tests respectively. * *p* < 0.05, ** *p* ≤ 0.01 (indicated *p* represent values from post-hoc tests), *N* = 5–7 (2 weeks); 4 (10 weeks); 4 (20 weeks) rats/ injury group, mean ± SEM.

We assessed changes in T- and B- cell frequencies, as well as in CD4^+^ and CD8^+^ T- cell subsets, due to cervical SCI in mandibular and deep cervical LNs and blood by flow cytometry (Fig 2). We did not observe any significant difference in the size of the draining LNs between the groups, as determined upon gross examination. Moreover, frequencies of T-cells (Fig 3a and 3b), and their CD4^+^(Fig 3a and 3d), or CD8^+^ (Fig 3e and 3f) subsets, and frequencies of B- cells (Fig 3g and 3h) were the same between the sham and SCI group for all time points of the study. In both injury groups, we found time-dependent changes in CD4^+^ and CD8^+^ T-cell subsets in mandibular LNs (Fig 3c and 3e) as well as alterations to B cell frequencies in mandibular (Fig 3g) and deep cervical LNs (Fig 3h). Specifically, we observed a significant decrease for CD4^+^ T-cells in mandibular LNs from 2 to 20 weeks in shams (*p* = 0.0003) and SCI rats (*p* = 0.019) (Fig 3c). On the other hand, CD8^+^ T- cells in mandibular LNs increased (Fig 3e) in shams (*p* = 0.002) and SCI rats (*p* = 0.014). Given the reverse relationship of CD4^+^ and CD8^+^ T-cell frequencies, we also estimated changes in CD4^+^: CD8^+^ ratios between 2 and 20 weeks, and found a significant decline in shams (*p* = 0.004) and SCI rats (*p* = 0.019). Moreover, B- cell frequencies decreased in mandibular (Fig 3g) and deep cervical (Fig 3h) LNs between 2 and 20 weeks of injury in both shams (*p* = 0.001 for mandibular and *p* = 0.01 for deep cervical LNs) and SCI rats (*p* = 0.033 for mandibular LNs; *p* = 0.01 for deep cervical LNs). Lastly, there were no time-dependent changes in CD4^+^ and CD8^+^ subset frequencies in deep cervical LNs (Fig 3d and 3f). Taken together, these data indicate that there was no effect of cervical SCI on T- and B- cell composition in the mandibular and deep cervical LNs. Also, factors related to post-operation duration or age (rather than SCI) may contribute to lowered CD4/CD8 ratios and B- cell frequencies in SCI and sham rats.

### Circulating T- and B- cell frequencies declined following cervical SCI

**Fig 4:**
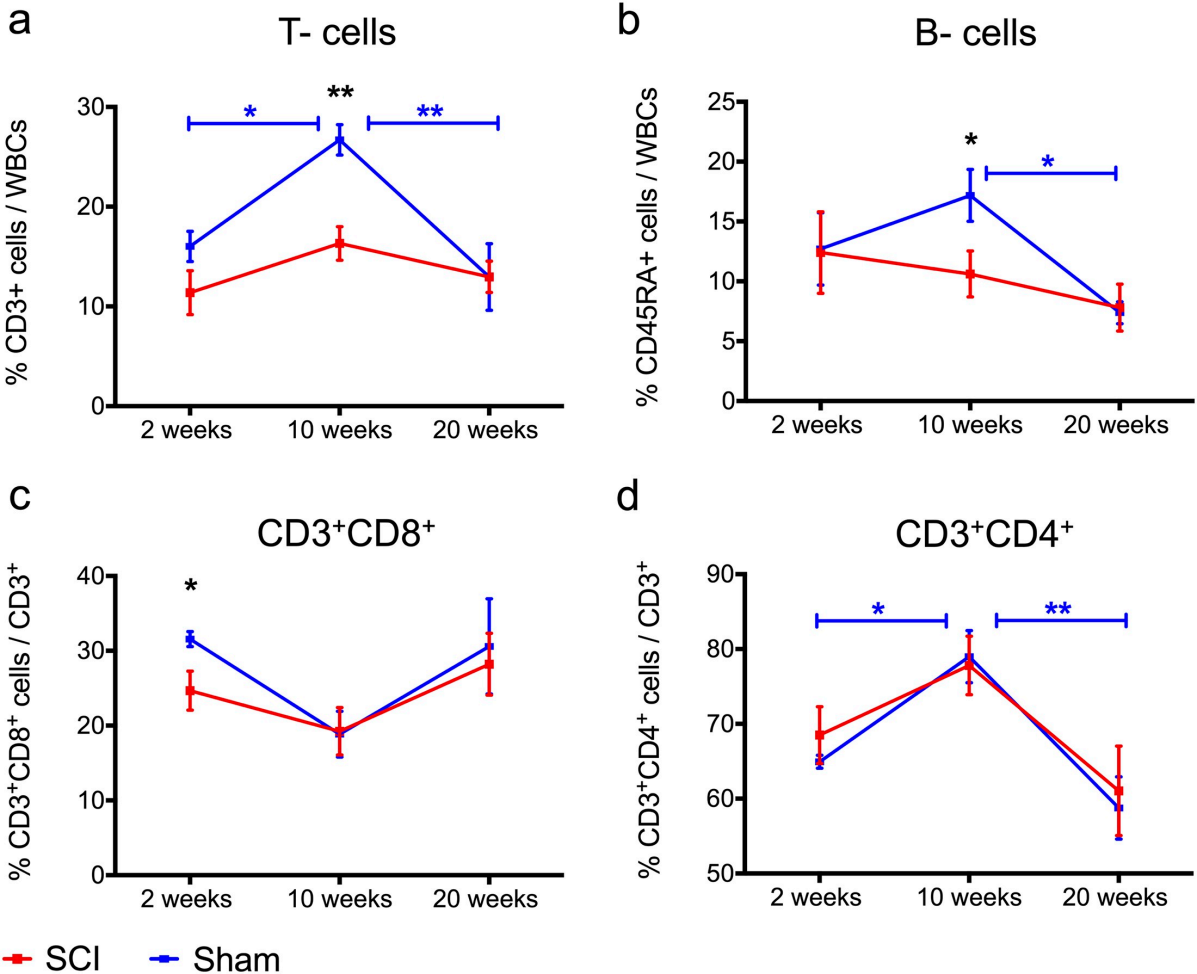
Significant decline in circulating T- and B-cell frequencies after cervical SCI compared to sham-operated rats. (a-b) T- and B-cell frequencies in rats with cervical SCI were significantly lower than in shams at 10 weeks post-injury. These frequencies did not change with time post-cervical SCI, whereas in shams T- and B- cells exhibited time-dependent changes; with (a) T- cells marking a significant increase from 2 to 10 weeks and a drop from 10 to 20 weeks, whereas (b) B- cell proportions declined from 10 to 20 weeks p.i. (c) CD8^+^ T- cell frequencies dropped at 2 weeks post cervical SCI, compared to shams, but no overall time-dependent changes were observed within each injury group. (d) CD4+ T- cells were similar between injury groups, although only CD4^+^ T- cells in sham animals showed time-dependent changes; with a significant increase from 2 to 10 weeks and a decrease from 10 to 20 weeks. For all data Independent Student’s t-tests were performed to analyze differences between sham and cervical SCI groups at each time point. Within each injury group, one-way ANOVA with Bonferroni post hoc tests between weeks was conducted to compare time-dependent changes. * *p* < 0.05, ** *p* < 0.01 (black * refers to difference between sham and SCI, blue and red * refer to time-dependent difference within the SCI or sham group following post-hoc testing, respectively); *N* = 5–7 (2 weeks), 6 (10 weeks), 7 (20 weeks) rats/ injury group, mean ± SEM.

Detecting significant changes to T- and B- lymphocytes in an accessible tissue such as blood can be of high diagnostic value in patients with a SCI. Here we assessed changes in circulating T- and B-lymphocytes after cervical SCI as compared to matched sham controls at 2, 10 and 20 weeks after injury. When comparing rats with SCI and their sham counterparts, we found T- and B- cell frequencies were unaffected at 2 weeks. However, at 10 weeks of injury, T cell frequencies declined by 39% (*p* = 0.001) and B cells by 38% (*p* = 0.047) compared to shams (Fig 4a and 4b). By 20 weeks of injury, these frequencies returned to levels similar to shams (Fig 4a and 4b). Moreover, CD8^+^ T-cells declined by about 22% (*p* = 0.041) compared to the sham group at 2 weeks, but returned to normal values at later time points (Fig 4c). The CD4^+^ T-cell subset was similar between groups and across all time points of the study (Fig 4d). When data were analyzed for time-dependent changes within each injury group, we found significant changes in T-, B- cells and CD4+ T-cells in sham rats, but not in rats with SCI. Specifically, T-cell frequencies in sham rats increased by 67% (*p* = 0.038) from 2 to 10 weeks and declined by 51% (*p* = 0.004) from 10 to 20 weeks (Fig 4a). B- cell frequencies in sham rats decreased by 57% (*p* = 0.013) from 10 to 20 weeks (Fig 4b). CD4+ T- cells increased by approximately 21% (*p* = 0.03) from 2 to 10 weeks and declined by approximately 26% (*p* = 0.001) from 10 to 20 weeks (Fig 4d). Although a similar trend was seen in CD4+ T- cells of rats with SCI, these effects did not reach statistical significance (Fig 4d). Lastly, total circulating leukocytes were similar between the SCI and sham groups across all time points of the study (Table 1).

**Table 1:** Statistical results from comparisons between SCI and sham rats at 2, 10- and 20-weeks post-injury for differences in counts of total circulating leukocytes. Independent Student’s t-tests were performed to compare SCI and sham groups within each time point.

Blood leukocytes (10^3^/μI)				
Time Point	Mean	SD	N	P value
2 weeks SCI	9.27	3.07	7	0.5806
2 weeks Sham	8.81	1.57	7	
10 weeks SCI	7.69	1.31	6	0.8774
10 weeks Sham	7.91	3.17	6	
20 weeks SCI	6.43	1.94	5	0.7281
20 weeks Sham	7.07	1.76	6	

Taken together, we found that blood T and B-cell proportions were affected after cervical SCI at various time points after injury; during the subacute phase (2 weeks) a decline in CD8^+^ T cell frequencies was found, whereas during the chronic phase of injury (10 weeks) T- and B-cell frequencies decreased. In the late chronic phase of the study (20 weeks), SCI rats had normal T-and B-cell frequencies. Lastly, T-, B- cells and CD4+ T- cells declined significantly between 10 and 20 weeks in sham rats, although they were unaltered in the SCI group.

### Spleen/body weight ratio increased following cervical SCI

**Fig 5:**
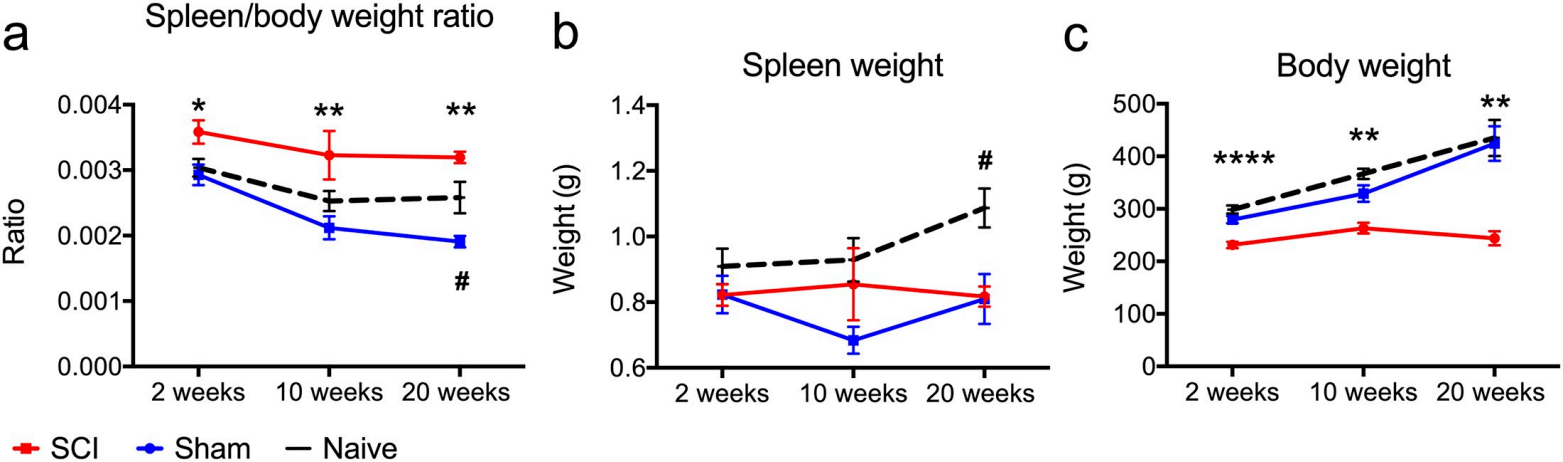
Larger spleen/body weight ratio following cervical SCI. (a) The spleen/body ratio was significantly higher in rats with cervical SCI compared to shams at all time points of the study. Also, when compared to the naive group, rats that received a sham injury had a smaller spleen/body weight ratio at 20 weeks. (b) Absolute spleen weight was not different between the cervical SCI and sham group. Also, the naive group had significantly larger spleen masses compared to the sham group at 20 weeks. (c) Rats with cervical SCI had significantly lower body weight compared to their sham counterparts at all time points of the study. There was no significant difference in body weight between sham and naive rats. One-way ANOVA with Bonferroni post hoc test was conducted to compare groups within each time point. * Indicates significant difference between sham and cervical SCI groups, whereas ^#^ indicates significant difference between sham and naive groups. */^#^ *p* < 0.05, ** *p* < 0.01, *** *p* < 0.001, **** *p* < 0.0001 (*p* indicates values for post-hoc tests), *N* = 6–12 (2 weeks); 6–8 (10 weeks); 4–6 (20 weeks) rats/group, mean ± SEM.

Splenic atrophy suggests compromised immune function in humans [22]. Mice with high-level thoracic SCI have been shown to have reduced spleen size and suppressed immune functions [10, 23] due to sympathetic dysregulation of the spleen, occurring in high-level but not in lower-level thoracic SCI. In line with these findings, we hypothesized that rats with cervical SCI would have smaller spleens compared to their sham counterparts. To test this hypothesis, we recorded spleen weights at 2, 10 and 20 weeks post-cervical SCI and compared them to time-matched shams. Additionally, age-matched naïve rats were included to control for age-dependent effects on spleen size [24]. Rats with cervical SCI were found to have a larger spleen/body weight ratio compared to shams at all time points of the study (Fig 5a) (*p* < 0.00001, Two-way ANOVA). When absolute spleen mass was considered, there was no difference between the cervical SCI and sham group in all time points of the study (Fig 5b) (*p* = 0.26, Two-way ANOVA). However, rats with SCI had substantially lower body weight than their sham counterparts (Fig 5c) (*p* < 0.0001, Two-way ANOVA), which may affect spleen size [25, 26]. Thus, spleen/body weight mass was selected as an ideal metric of spleen size changes in this study. For each injury group, the spleen/body weight ratio remained constant across all time points. In the naïve group, the average spleen/body weight ratio had an average value of 0.27%, which is consistent with previous reports in normal rats [27].

Taken together, these data show cervical SCI results in splenomegaly in the rat, which suggests a state of splenic hyperfunction.

### Serum IgG and IgM levels declined sub-acutely, but IgM immunoglobulin increased in the chronic phase of cervical SCI

**Fig 6:**
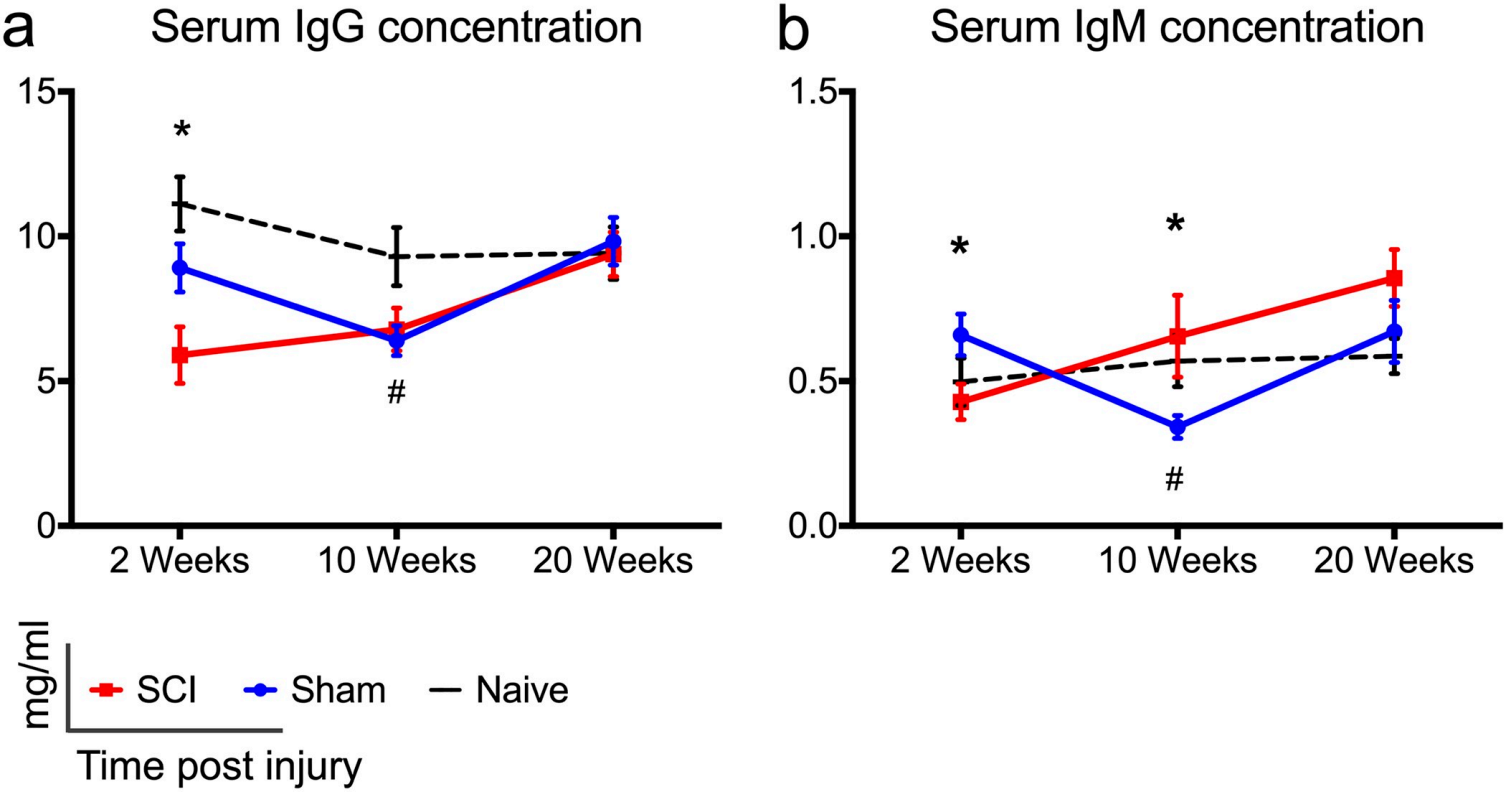
Changes in serum IgG and IgM immunoglobulin levels following cervical SCI. (a, b) Serum IgG and IgM levels declined significantly at 2 weeks post-cervical SCI compared to shams. In rats with cervical SCI, IgG immunoglobulin normalized to sham-levels at later time points. IgM levels were statistically higher in the SCI groups compared to shams at 10 weeks of injury. Naïve rats had significantly higher levels of IgG and IgM antibodies compared to sham rats at 10 weeks. One-way ANOVA with Holm-Sidak’s post-hoc test was performed for IgG data (2 weeks), and Kruskal Wallis analysis with Dunn’s multiple comparisons post-hoc tests were performed for all other data. * Indicates significant difference between sham and SCI groups, whereas ^#^ indicates significant difference between sham and naïve groups. */^#^ *p* < 0.05, *N* = 9–10 (SCI); 7–8 (Sham); 5–7 (Naive); rats/group, mean ± SEM.

Research from our group and others suggests that cervical SCI and high-level thoracic SCI result in impaired antibody responses. For example, high-thoracic SCI has been shown to result in reduced T- dependent antibody responses [9, 10], and we recently found lower levels of total serum IgG and IgM immunoglobulin at 2 weeks post- C7/T1 SCI in rats [15]. However, whether impaired antibody responses persist in the chronic phase of cervical SCI remains unknown. Here we found lower levels of IgG (Fig 6a) (*p* = 0.025) and IgM immunoglobulin (Fig 6b) (*p* = 0.03) in rats with cervical SCI compared to sham animals at 2 weeks post injury, confirming our previous observations [15]. During the chronic time points of our study (10 and 20 weeks p.i.) IgG concentration returned to sham-comparable levels (Fig 6a), whereas IgM levels were higher in rats with SCI (Fig 6b); a difference that reached significance at 10 weeks (*p* = 0.017). Lastly, sham rats had lower levels of IgG and IgM immunoglobulin compared to naïve rats at 10 weeks (Fig 6a, *p* = 0.038), (Fig 6b, *p* = 0.05).

Taken together, these data show that following cervical SCI the levels of circulating antibodies decreased during the subacute phase of injury, whereas IgM immunoglobulin increased in the chronic phases of injury. Sham injury alone may have contributed to lowered levels of IgG and IgM immunoglobulin at 10 weeks.

## Discussion

Over half of clinical SCIs occur at the cervical level (1), but the effects of cervical SCI on the peripheral immune response remain unknown. In this study, we assessed the effect of cervical SCI on peripheral adaptive immunity in Wistar rats by profiling T- and B- cell frequencies in the blood and draining LNs, as well as levels of serum IgG and IgM immunoglobulin, and spleen size. These assessments were done at 2, 10 and 20 weeks after injury, representing the subacute, chronic and late chronic phases of SCI, respectively. Our data showed evidence of limited immunosuppression that returned to normal by 20 weeks post-injury. In particular, we saw lowered immunoglobulin levels at 2 weeks of SCI, coupled with declined CD8^+^ T cell frequencies in the blood, as compared to sham rats. Moreover, at 10 weeks after SCI, circulating T- and B- cell frequencies declined. Also, at 10 weeks we observed a significant increase in IgM immunoglobulin. Although all these effects were normalized by 20 weeks post-SCI, the spleen/body weight ratio was higher in rats with SCI throughout the study course. Below we discuss the novelty, clinical relevance and importance of these findings for furthering our understanding of SCI pathophysiology.

This is the first report on the effect of cervical SCI on T- and B- cell frequencies of mandibular and deep cervical LNs, which drain the spinal cord [28, 29] and brain [30, 31], and are responsible for fighting upper respiratory tract infections in humans [32]. Tracing experiments have indicated that spinal cord antigens normally are drained in mandibular, deep cervical and lumbar LNs [29, 33, 34] following their uptake by meningeal lymphatics and their exit via nerve roots [35]. As such, tracers have been injected in the ventricles [36], atlanto-occipital [29], or thoracolumbar [34, 37] region of the SAS, but not at the spinal cord cervical level of the SAS. As CSF [33, 34] and parenchymal [38] drainage from the spinal cord may vary along its rostro-caudal axis, it thus remained unclear whether antigens from the cervical spinal cord are also drained in mandibular, deep cervical and lumbar LNs. To this end, we performed tracing experiments by injecting india ink, a 20–50 nm particle size dye [39], intrathecally at the C7 level. Based on our observations, mandibular and deep cervical LNs were found to drain the cervical spine, and were further assessed for changes in the composition of T- and B-cell frequencies post- C7/T1 SCI. We did not assess changes in cell composition of lumbar LNs, as we found no drainage from the SAS of the cervical spine into these LNs. Interestingly, there is controversy whether the lumbar LNs receive antigenic drainage from the spinal cord. Vega and Jonakait argue that drainage into lumbar LNs is seen in experiments where the dye infusion rate in the SAS is higher than the physiological CSF flow rate (0.5 μl/min) [28]. In our experiments, india ink was infused intrathecally at a flow rate 15 μl/min. Yet, lumbar LNs were stained in only one animal (out of 12), suggesting that CSF flow rate may have some, but not a crucial effect on dye drainage into lumbar LNs. Relatedly, it should be noted that the efficiency of fluid uptake by lymphatic vessels depends on several other factors including the anesthetic used, the depth and duration of anesthesia, respiration and blood pressure [34].

Our data showed no significant changes in T- and B- cell composition of mandibular and deep cervical LNs after cervical SCI. Research in animal models of thoracic SCI has shown that the frequencies and/or function of T- and B- cells in the CNS-draining LNs is downregulated in an injury level-dependent fashion, due to loss of sympathetic nervous system (SNS) control and excessive activation of the hypothalamic pituitary adrenaline (HPA) axis. For instance, in a rat T8 injury model, increased serum corticosterone (CORT) levels due to HPA activation were suggested to cause a reduction in LN B- and cytotoxic (CD8+) T- cell frequencies without affecting their function [40]. Also, after T4 injury, high systemic norepinephrine (NE) levels due to SNS dysregulation were linked to diminished antibody responses against spinal CSF antigens in mandibular and deep cervical LNs early on after injury [41]. In rats with T1 SCI, the proliferative capacity of T- cells in deep cervical LNs declined for up to 30 days after injury [9] (although their T-cell composition was not characterized). Taken together, research in thoracic SCI models suggests that the intensity and duration of immune system compromise in draining LNs depends on the level and severity of injury. We expand these observations by showing that following C7/T1 SCI, the T- and B- cell composition of mandibular and deep cervical LNs does not change between the subacute (2 weeks) to late chronic (20 weeks) injury phases. It should be noted that factors other than level of injury, such as species and strains used to model SCI [42–44], can impact immunological responses and explain potential differences between our study and thoracic SCI studies.

While here we looked at lymphatic drainage of the cervical spinal cord in the naïve state, future studies should characterize lymphatic drainage following SCI. Ageing was shown to impair brain lymphatic drainage in mice, and to reduce lymphatic vessel diameter and lymphatic coverage [45]. In parallel, ablation of lymphatic vessels in a mouse model of Alzheimer’s disease resulted in reduced amyloid-β clearance [45]. Taken together these studies suggest that lymphatic function in the CNS can be modified by factors such as ageing, and that efficiency of lymphatic drainage can affect CNS disease outcomes [31, 46]. Thus, it is possible that lymphatic vessels in the spinal cord are damaged due to trauma, resulting in diminished drainage of antigens of the injured parenchyma and CSF. Reduced spinal cord drainage could explain the absence of significant changes in the immune cell composition of the draining LNs following SCI, in the present study. Therefore, future studies should assess whether SCI damages lymphatic vessels resulting in reduced drainage, and whether modifying lymphatic drainage could improve disease outcomes.

Splenic immune cell phenotypes and function are critically affected after SCI [47]. Studies in thoracic SCI have consistently shown that splenic function is compromised in an injury-level dependent fashion; whereby high-thoracic injuries cause substantial lymphocyte apoptosis, as well as impaired immune function and smaller spleen size [7, 10, 23, 48]. Lymphocyte apoptosis and immune compromise in the spleen are caused by CORT and NE, which increase early after high level thoracic SCI [23, 48–50], as well as in episodes of autonomic dysreflexia throughout the course of SCIs above T6 [51, 52]. However, changes in spleen size following cervical SCIs are not characterized as comprehensively as in thoracic SCIs.

We previously found that the splenic immune response is predominantly auto-reactive at 2 weeks post-C7/T1 SCI in rats, as shown by increased counts of antibody secreting cells and the development of an autoantibody response against spinal cord antigens [15]. Here, we extend these observations by showing that C7/T1 SCI results in splenomegaly–a marker of splenic hyperfunction–at all time points of our study, as indicated by higher spleen/body weight ratios. While splenomegaly at 2 weeks post-SCI is congruent with our previous results showing increased splenic autoreactivity at the same time point [15], the reasons for splenomegaly in the chronic time points of the present study (10 and 20 weeks) require further investigation. We previously found that SCI results in an augmented antibody response in the spleen, which was characterized by increased T-dependent antibody (IgG) responses at 2 weeks, and by expansion of IgM antibody secreting cells throughout the study course (2 to 20 weeks). Given that in the SCI group splenic IgM secreting cells were twice as many as in the sham group, we posit that the small increase in spleen weight seen in the SCI animals in the present study could be due to an expansion in IgM secreting cells.

Other studies have reported increased spleen/body mass ratios at 2 weeks of C6/C7 SCI in rats [8], as well as in humans with SCI. In particular, two studies found asymptomatic splenomegaly in a significant percentage of chronic patients with SCI, although their injury level was not specified [53, 54]. Of note, in our current study rats with SCI presented with significant body weight loss compared to matched shams; which led us to report changes in terms of the spleen/body mass ratio. However, contrary to rats, patients with SCI are at high risk for becoming obese or overweight [55]. Thus, the clinical significance of the spleen/body mass data presented here remains to be explored.

We found significant effects of cervical SCI on blood lymphocytes as well as IgG and IgM immunoglobulin. We showed that blood CD8+ T lymphocyte proportions declined at 2 weeks, and T- and B- cell frequencies declined at 10 weeks of C7/T1 SCI, as compared to sham rats. CD8+ T- cell frequencies are important to monitor in patients with SCI as their decreased frequencies and effector functions could compromise patients’ ability to mount effective immune responses against viral infections. Indeed, antiviral activity is suppressed after SCI in mice acutely [56] and chronically [57, 58], and the incidence of death due to pneumonia and influenza are higher in patients with SCI than in the general population [13]. Others have also reported declined CD8+ T-cell frequencies early after SCI in patients, which subsequently returned to normal values in the chronic stage of injury [59]. Increased levels of glucocorticoids post-SCI [such as cortisol in humans [59], or CORT in rodents [40, 56]] and NE [58] regulate CD8+ T- cells by inhibiting their proliferation [56] and function [56, 58].

In the early chronic phase of SCI (10 weeks p.i.) we found lowered T- and B- cell frequencies compared to sham controls. Similar observations have been reported in studies of patients with tetraplegia and paraplegia in the chronic stage. In particular, Pavlicek showed a decline in total blood lymphocytes [17], and Monahan showed lowered T- cell frequencies compared to able-bodied controls who were matched for age and sex [60]. Moreover, in Pavlicek’s study blood lymphocytes were shown to decline significantly with time after injury in patients with SCI, but this effect was less profound than in controls [17]. Similarly, we found a significant drop in CD4+ T cells and B- lymphocytes in sham rats between 10 and 20 weeks, but less so in rats with SCI. Moreover, sham rats showed a steep and significant increase in T cells and CD4+ T cells between 2 and 10 weeks, whereas in the SCI rats we did not observe significant differences for the same course of the study. While the increase in circulating T cells in sham rats may have resulted from the sham injury itself (characterized by extensive hemorrhage, severe skeletal muscle injury and resection of the bone of the spinal column) [61–63], the SCI rats seem to have lost the ability to mount a similar immunological response to the muscle and bone injury they incurred. Taken together, the changes in blood T- and B- lymphocytes observed here are largely in line with observations in SCI patients, suggesting that our rat C7/T1 SCI model has important clinical relevance for studying the effects of SCI on systemic lymphocytes.

Lastly, we detected lower total levels of serum antibodies at 2 weeks after cervical SCI, confirming our previous observations [15]. Declined levels of circulating IgG and IgM antibodies indicate compromised adaptive immunity and could result in suboptimal immune responses following microbial infections. Since we previously found increased counts of antibody secreting cells in the spleen at 2 weeks of cervical SCI [15], lowered serum immunoglobulin levels suggest that antibody synthesis in the bone marrow–another major source of serum immunoglobulin–could be downregulated post-SCI [64–66]. Indeed, in patients with SCI, bone marrow hematopoietic progenitor cells have impaired function and proliferative capacity, which could hamper antibody synthesis [67]. Of note, a study found increased levels of IgG after SCI and similar levels of serum IgM between patients with SCI and able-bodied controls, as measured at 31 days after injury [68]. However, these results are difficult to interpret as they were not corrected for age and gender, which greatly affect IgG and IgM levels [69–71].

The novel finding of high levels of circulating IgM immunoglobulin in the chronic phase of cervical SCI could be explained by our study showing increased IgM antibody secreting cells in the spleen during the same time points in C7/T1 SCI [15]. The role of this systemic IgM immunoglobulin increase in the outcomes of SCI remains to be explored. Circulating IgM immunoglobulin comprises the majority of naturally occurring antibodies, which were shown to maintain homeostasis against autoimmunity, infectious and other diseases [72]. Thus, it is possible that an increase in serum IgM could confer protection following SCI, by reducing the autoantigenic load and by enhancing clearance of lesional debris post-injury.

## Conclusions

In conclusion, this study demonstrates the effect of cervical SCI on peripheral adaptive immunity by profiling changes in T- and B- cells in spinal cord draining LNs and blood, as well as changes in spleen mass and levels of serum immunoglobulin. Our data show that cervical SCI causes disturbance to circulating lymphocytes and immunoglobulin levels between 2- and 10-weeks post-injury, while all these outcomes are normalized by 20 weeks. This finding is anticipated to guide future research establishing the duration of peripheral immune disturbance following SCI in patients, which will ultimately improve existing strategies for the administration of systemic immunosuppressive regimens post-SCI. Moreover, routine establishment of circulating lymphocytes and immunoglobulin levels will help identify patients in greater need for prophylactic vaccination. Research indicating that patients with SCI respond optimally to vaccines against influenza [73] and pneumococcus [74, 75] creates optimism that immunological protection is achievable with vaccination, despite disturbed immune functions resulting from SCI.

## Supporting information

S1 Data (XLSX)
